# Probiotic fruit beverages with different polyphenol profiles attenuated early insulin response

**DOI:** 10.1186/s12937-018-0335-0

**Published:** 2018-02-27

**Authors:** Jie Xu, Tommy Jönsson, Merichel Plaza, Åsa Håkansson, Martin Antonsson, Irini Lazou Ahrén, Charlotta Turner, Peter Spégel, Yvonne Granfeldt

**Affiliations:** 1 Department of Clinical and Experimental Medicine, Linköping University, Linköping, Sweden; 2 Center for Primary Health Care Research, Lund University, Skåne University Hospital, Malmö, Sweden; 3 Department of Analytical Chemistry, Physical Chemistry and Chemical Engineering, Faculty of Biology, Environmental Sciences and Chemistry, Universidad de Alcalá, Ctra. Madrid-Barcelona Km. 33.600, Alcalá de Henares, Madrid Spain; 4 Department of Chemistry, Lund University, Lund, Sweden; 5 Department of Food Technology, Engineering and Nutrition, Lund University, Lund, Sweden; 6 ProViva AB, Lunnarp, Sweden; 7 Probi AB, Lund, Sweden

**Keywords:** Bilberries, Blackcurrant, Mango, Rose hip, Beetroot, Postprandial insulin response

## Abstract

**Background:**

Consumption of polyphenol-rich fruits and vegetables may improve postprandial glucose and insulin levels and hence promote well-being. Previously it has been observed that consumption of bilberry decreases the postprandial insulin demand. The intention with the present study was to compare the impact of different supplements with various polyphenol profiles, on the postprandial glucose and insulin responses in healthy young adults.

**Methods:**

In a randomized, controlled, crossover study the postprandial glycemic and insulin responses were observed in eleven healthy adults after intake of five different beverages containing bilberry (European blueberry), blackcurrant, beetroot, mango and rose hip, respectively; all drinks were enriched with the same composition of fermented oatmeal and probiotics. The control was a glucose drink. The profile and content of the polyphenols in the different beverages were determined by HPLC-DAD analysis. The antioxidative capacity of the different beverages were measured by TEAC and DPPH assays.

**Results:**

Beverages containing bilberry, blackcurrant, mango or rose hip significantly attenuated the early postprandial insulin response (0–90 min), but showed no effect on glucose response. Drinks with bilberry or rose hip reduced the insulin response from the very early phase (0–30 min), and had significantly lower insulin index compared with the control. The efficiency of the bilberry and rose hip to decrease early postprandial insulin responses correlated with higher phenolic contents.

**Conclusions:**

Supplements with bilberry, blackcurrant, mango or rose hip in the tested probiotic and oatmeal enriched beverage attenuated early-phase insulin response, but had no effect on the postprandial glycemic response. The improved ability of bilberry and rose hip to lower the very early phase of insulin response seems to be due to a higher phenolic content.

**Trial registration:**

The study was retrospectively registered at ClinicalTrials.gov with number NCT03159065.

**Electronic supplementary material:**

The online version of this article (10.1186/s12937-018-0335-0) contains supplementary material, which is available to authorized users.

## Background

Postprandial hyperglycemia contributes to the development of cardiovascular diseases and type 2 diabetes [1, 2]; both diseases lay burden on the healthcare systems, globally [3]. Postprandial hyperglycemia is a condition that can be improved through lifestyle and diet modifications. In functional food research, dietary polyphenols have shown potential to improve the postprandial glucose and insulin responses and promote well-being via several postulated underlying mechanisms: 1) inhibition of carbohydrate digestion and the subsequent glucose absorption, 2) stimulation of insulin secretion from pancreatic β-cells, 3) modulation of hepatic glucose output, 4) activation of insulin receptors and glucose uptake in insulin-sensitive tissues, and 5) modulation of intracellular signaling pathways and gene expression [4–6].

Fruits, berries, vegetables, tea and coffee are the common sources of dietary polyphenols [7]. Decreased postprandial blood glucose and insulin responses have been reported in healthy adults after consumption of freeze-dried fruits (apple peel, blackberry, blackcurrant and strawberry) with green tea [8], beetroot juice [9], and blackcurrants and lingonberries [10]. In healthy adults, an attenuated postprandial insulin response, but not glucose response was also reported in some studies with extract of baobab fruit [11] and berries [12]. Similar effects were observed in obese and insulin-resistant male volunteers after intake of black tea, but not after intake of beetroot juice [13]. Although the polyphenol-rich fruits and vegetables have showed improving effects on postprandial glycemic and/or insulin responses, the efficacy of the dietary polyphenols in improving postprandial glucose and insulin responses seems to be influenced by multiple factors such as source of polyphenols, dose and sort of polyphenols. For example, the postprandial response was more efficiently decreased after consumption of blackcurrant than of lingonberries [10]. It was speculated that the difference in polyphenol compositions between the two berries could be the contributing factor. However, in another study performed by the same group, no correlation could be found between the insulin lowering effect and the polyphenol composition of the berries [12].

In a previous study [14], we observed that intake of a drink containing oatmeal fermented by the probiotic strain *Lactobacillus plantarum* 299v and supplemented with bilberry (European blueberry) induced a lower postprandial insulin response compared with the white bread control. The lowered acute insulin demand was more profound with higher dose of bilberries. In addition, a corresponding drink supplemented with rose hip decreased the insulin response to some extent, however, not to a statistically significant level. Thus, it is important to clarify the relationship between dietary polyphenols and insulin response.

In the present study, we hypothesized that polyphenol rich beverages enriched with fruits (bilberry, blackcurrant, rose hip and mango) and vegetable (beetroot) can attenuate postprandial glycemic and insulin responses. The presence of a probiotic strain with polyphenol splitting ability in the drink may enhance the physiological availability of the phenolics [15]. The acute postprandial glycemic and insulin responses as well as the polyphenol content and antioxidative capacity of the tested beverages were measured. The relation between the polyphenol content and the postprandial glycemic and insulin responses were examined with multivariate data analysis.

## Methods

### Study design and participants

The study was a randomized, controlled, crossover study with the aim to investigate and compare the postprandial glycemic and insulin responses after consuming five different probiotic fruit or vegetable beverages. Twelve healthy young adults, 6 men and 6 women (median age 23.5 years with interquartile range [IQR] 23–29.5 years) with body mass index (BMI) 24.3 ± 2.4 kg/m^2^ (mean ± SD) were recruited in the study. The inclusion criteria were healthy adult individuals (18–65 years) with no medication, no diagnosed allergy, and having BMI between 20 and 30 kg/m^2^ with a stable body weight (less than 5% weight change in the last three months before the inclusion).

The study was performed at the clinical research center at Skåne University Hospital (Lund, Sweden) and was completed within 6 weeks. Every participant visited the study center once a week (~ 3 h per visit; about every 7th day). The evening (9–10 pm) before each test, the participants were asked to eat a standardized meal consisting of two pieces of white bread and were thereafter only allowed to drink a small amount of water. On the test day, venous blood from the left arm was sampled and within the following 15 min participants were given one of the five test products or the reference drink, in a randomized order. A randomized table with weekly intervention for each individual was generated before the start of the study using an online random number generator (https://www.random.org). Thereafter, 3 and 3.5 ml of blood samples were taken at 15, 30, 45, 60, 90 and 120 min to analyze postprandial glucose and insulin responses, respectively. The beverage was served from newly opened packages.

### Study products

The test products with the trade name ProViva® were provided by ProViva AB (Solna, Sweden). It is a fruit drink containing fermented oatmeal and live *Lactobacillus plantarum* 299v (DSM 9843; Probi AB, Lund, Sweden). The test products were supplemented with different fruits or vegetables: 1) 10% bilberry (Bilberry), 2) 25% blackcurrant (Blackcurrant), 3) 15% beetroot (Beetroot), 4) 6.5% mango (Mango), and 5) 14% rose hip (Rose hip). The amount of the beverages was standardized to contain 30 g of the available carbohydrates. The amount of the test products served to the participants were: Bilberry, 244 g; Blackcurrant, 262 g; Beetroot, 272 g; Mango, 288 g; and Rose hip 285 g. The reference glucose drink was prepared by dissolving 30 g of the glucose in 270 g of water. To note, we also included another control drink based on only fermented oatmeal with added sugar which was later excluded for analysis when we realized that the added sugar was not in correct amount.

### Analyses of blood samples

Venous blood samples were taken through a venous catheter in the morning between 09:00 and 12:00 h after fasting for 10 h. Whole blood were drained into 5 ml of serum and plasma separation tubes. Plasma glucose and serum insulin were analyzed by standard methods at the Department of Clinical Chemistry, Skåne University Hospital, Lund, Sweden.

### Viable count of lactobacilli

Samples taken from the five test products were serially diluted, and 100 μl from each dilution were plated on MRS agar and incubated anaerobically for 24–72 h at 37 °C prior to counting of colony forming units (CFU).

### Sample preparation for analysis of phenolic compounds and antioxidant capacity determination

The five test beverages were centrifuged for 10 min. The supernatant was then filtrated through Teflon filter (0.2 μm) (VWR International, West Chester, PA, USA) prior to the analysis without further clean up.

### Analysis of phenolic compounds during shelf life

Batch and inter-day variations were investigated for the five test products when stored in unopened packages at + 4 °C. Two different batches of the four test products (Bilberry, Blackcurrant, Mango and Rose hip) were analyzed during the study time. The first batch was analyzed on the first day of the intervention and 4 weeks later (shelf life). The second batch was analyzed in the week 5 and 6 (completion of the study). Three batches were analyzed for Beetroot. The first batch was analyzed on the first day of the intervention and 2 weeks later (shelf life). The second batch was analyzed in the week 3 and 5. The third batch was analyzed in week 4 and 6 of the study. Analysis of phenolic compounds in the five probiotic drinks was determined by HPLC-DAD method according to a previous work [16] with some modifications. Separation was carried out with porous-shell fused core Ascentis Express C18 analytical column (150 mm × 2.1 mm, 2.7 μm) from Supelco (Bellefonte, PA, USA). The mobile phases consisted of (A) water with 5% of formic acid, and (B) methanol with 5% of formic acid in a gradient elution analysis programmed as follows: 0 min, 5% (B); 0–5 min, 5% (B); 5–35 min, 40% (B); 35–40 min, 40% (B); with 10 min of post-time for column conditioning at a flow rate of 300 μl/min. The column temperature was set at 50 °C, the injection volume was 2 μl and the vial tray was held at 4 °C. Three wavelengths 280 nm, 350 nm and 520 nm were selected for identification of phenolic acids, flavonols and anthocyanins/betalains, respectively.

### Total phenolic content

The total phenolic content of the test products was determined by the Folin-Ciocalteu (FC) assay [17]. The volume of the reaction mixture was miniaturized to 1 ml. 10 μl of sample were mixed, to which 50 μl of undiluted Folin-Ciocalteu reagent was subsequently added. After 1 min, 150 μl of 2% (*w*/*v*) Na_2_CO_3_ and 790 μl of water were added. After 2 h of incubation at 25 °C, 300 μl of the mixture was transferred into a well of the microplate, the absorbance was measured at 760 nm in a microplate spectrophotometer reader (Multiskan GO, Thermo Fisher, Germering, Germany) and compared to a gallic acid calibration curve (0.025–2.000 mg/ml) elaborated in the same manner. Three different packages of each beverage were analyzed and each package was analyzed in triplicate. The total phenolic content was estimated as gallic acid equivalents (GAE), expressed as mg gallic acid/ml of test drink.

### Total antioxidant capacity

Trolox equivalent antioxidant capacity (TEAC) assay and DPPH assay were used to determine the total antioxidant capacity.

The TEAC assay employed was described by Re et al. [18] with some modifications. ABTS radical cation (ABTS^**·**+^) was produced by reacting 7 mM ABTS with 2.45 mM potassium persulfate and allowing the mixture to stand in the dark at room temperature for 12–16 h before use. The aqueous ABTS^**·**+^ solution was diluted with 5 mM phosphate buffer (pH = 7.4) to an absorbance of 0.70 (± 0.02) at 734 nm. Ten microliters of test beverages (four different dilutions) were added to 1 ml of diluted ABTS^**·**+^ radical solution. After 50 min at 30 °C, 300 μl of the mixture were transferred into a well of the microplate, and the absorbance was measured at 734 nm in a microplate spectrophotometer reader (Thermo Fisher). Trolox was used as a reference standard and results were expressed as TEAC values (mmol Trolox/l of test beverage). These values were obtained from at least four different dilutions of each test product in the assay giving a linear response between 20 and 80% of the initial absorbance. Three different packages of each product were analyzed and each package was analyzed in triplicate.

The DPPH radical scavenging assay was used [19]. Briefly, a solution was prepared by dissolving 23.5 mg of DPPH in 100 ml of methanol. This stock solution was further diluted with methanol 1:10. Both solutions were stored at 4 °C until use. Four different dilutions of each beverage were tested. 25 μl of these solutions were added to 975 μl of DPPH diluted solution to complete the final reaction medium (1 ml). After 4 h at room temperature, 300 μl of the mixture were transferred into a microplate well and the absorbance was measured at 516 nm in a microplate spectrophotometer reader (Multiskan GO, Thermo Fisher). DPPH-methanol solution was used as a reference sample. The DPPH concentration remaining in the reaction medium was calculated from a calibration curve. Three different packages of each beverage were analyzed and each package was analyzed in triplicate. The results are presented as DPPH radical inhibition (%).

### Data treatment and statistical analysis

All univariate statistical analyses were conducted in RStudio Version 0.99.902 [20]. The incremental areas under the curves (IAUCs) were calculated for blood glucose and serum insulin using the trapezoidal rule and ignoring the area below the baseline. Glycemic index (GI) and insulin index (II) were calculated from the IAUCs obtained after the intake of test products divided by the IAUCs obtained after intake of the reference glucose drink with each participant as his/her own control. GI and II were calculated for both 0–90 min and 0–120 min. In a few cases of hemolysis, missing data were substituted by mean values calculated from the same time point but different weeks. Three participants did not complete one test occasion each (one for mango, one for bilberry and one for blackcurrant) and were hence excluded from the data analysis of the corresponding group. Incremental blood glucose and serum insulin data were not normally distributed, thus Skillings-Mack test (equivalent to Friedman test but with the possibility to handle missing data) [21] was used to detect differences between the six beverage groups. When significant differences were detected, Conover post hoc test was performed for pairwise comparisons with false discovery rate (FDR) *P*-value adjustment. Comparisons of glycemic and insulin indices were performed using Wilcoxon signed rank test and *P* values were corrected for multiple comparisons. Multivariate data analyses were performed with SIMCA-P+ 12.0.1 (Umetrics, Umeå, Sweden). *P* < 0.05 was considered statistically significant.

The study was approved by the Ethics Committee of the Faculty of Medicine at Lund University, Sweden (Dnr 2014/435).

## Results

One participant discontinued due to personal reasons after two test occasions, and was removed from the study. Thus the study is based on eleven participants.

### Viable count

The lactobacilli counts in the five probiotic drinks were stable during the storage period, and between the different batches (3–5 × 10^7^ CFU/ml).

### Glycemic response

**Fig. 1:**
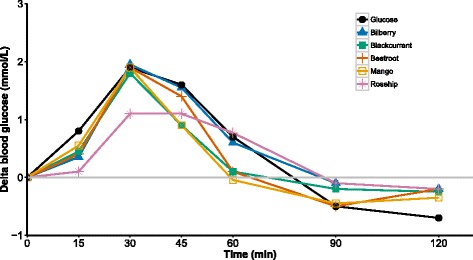
Incremental blood glucose response curve during the two-hour postprandial period. The change in blood glucose concentration was calculated by deducting the basal level from the blood glucose level measured after intake of the test drinks and was represented as delta blood glucose. Median values were plotted

The incremental blood glucose response after intake of the Bilberry, Blackcurrant, Beetroot, Mango, Rose hip products and the reference glucose drink are shown in Fig. 1. No significant differences regarding the incremental blood glucose concentrations were found between the groups at any of the time points, and the same was true for the glycemic responses measured by IAUCs (Table 1).

**Table 1:** Incremental area under two-hour blood glucose response curve

Tested products	IAUC(0–15 min) (mmol min/L)	IAUC(0–30 min) (mmol min/L)	IAUC(0–45 min) (mmol min/L)	IAUC(0–60 min) (mmol min/L)	IAUC(0–90 min) (mmol min/L)	IAUC(0–120 min) (mmol min/L)
Glucose (*n* = 11)	6.0 (3.4–7.9)	26.3 (17.6–36.0)	45.0 (37.6–67.5)	63.0 (44.7–91.5)	71.7 (48.9–112.1)	71.7 (48.9–117)
Bilberry (*n* = 10)	2.6 (0.8–4.5)	19.1 (13.5–24.8)	44.3 (33.8–52.5)	50.6 (45.8–73.5)	58.6 (47.5–82.5)	61.5 (47.5–82.5)
Blackcurrant (n = 10)	3.4 (1.5–6.8)	23.3 (13.5–30.0)	44.6 (31.5–63.8)	55.5 (39.0–71.3)	57.6 (40.5–101.3)	57.6 (45.1–111.8)
Beetroot (n = 11)	3.0 (0.8–3.0)	20.3 (15.8–22.1)	40.5 (33.0–64.9)	53.3 (34.3–93.4)	54.2 (34.3–118.6)	54.2 (35.0–123.2)
Mango (n = 10)	4.1 (0–6.0)	24.4 (11.3–29.3)	42.2 (28.5–53.3)	46.7 (33.8–66.1)	50.0 (34.0–66.1)	54.0 (34.0–66.1)
Rose hip (n = 11)	0.8 (0–2.3)	12.0 (5.2–16.9)	29.6 (16.8–42.0)	46.8 (26.7–65.6)	48.0 (29.9–88.2)	51.3 (30.0–96.4)

Data are presented as median (IQR)

### Insulin response

**Fig. 2:**
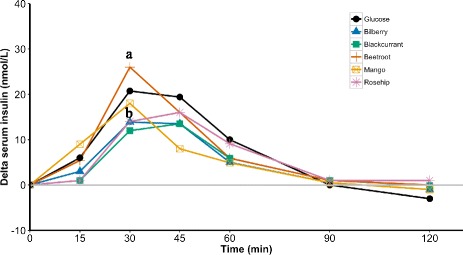
Incremental serum insulin response curve during the two-hour postprandial period. The change in serum insulin concentration was calculated by deducting the basal level from the insulin level measured after intake of the test drinks and was represented as delta serum insulin. Median values were plotted. Median values with different letters denote significant differences between Beetroot and Bilberry, and between Beetroot and Rose hip (*P* < 0.05)

The incremental serum insulin response after intake of the test products and the reference glucose drink are shown in Fig. 2. A significant difference (*P* = 0.03) in the serum insulin concentrations between the six groups was seen after 30 min. Pairwise comparisons showed that Bilberry and Rose hip groups had significantly lower increase in serum insulin levels compared with the Beetroot group (*P* = 0.02 and P = 0.02, respectively). Blackcurrant also showed a tendency of lower serum insulin elevation compared with Beetroot (*P* = 0.05). No significant differences were detected between the groups at other time points.

Comparisons of the IAUCs at different time periods are presented in Table 2. The IAUCs (0–30 min) were significantly different (P = 0.03) between the groups. Pairwise comparisons showed that Bilberry and Rose hip had significantly lower values of IAUCs compared to the reference Glucose group (*P* = 0.032 and P = 0.032, respectively). Additionally, in comparison to Bilberry and Rose hip, Beetroot and Mango showed significantly higher values of IAUCs (Beetroot vs. Bilberry, *P* = 0.033; Beetroot vs. Rose hip, P = 0.033; Mango vs. Bilberry P = 0.032; and Mango vs. Rose hip, P = 0.032). A similar pattern was observed for IAUCs (0–45 min). A significant difference was detected in the overall comparisons (*P* = 0.01). Compared to Glucose, Bilberry and Rose hip showed significantly lower IAUCs (*P* = 0.007 and P = 0.007, respectively). In addition, Blackcurrant also showed a lower value of IAUC compared with the reference Glucose although slightly above the statistical significance threshold (*P* = 0.053). Moreover, in comparison to the Bilberry and Rose hip, Beetroot showed a significantly higher value of IAUC (P = 0.007 and P = 0.007, respectively). In the period of 0–60 min, there was a significant difference among the groups (*P* = 0.019). Pairwise comparisons showed that Bilberry had a significantly lower value of IAUC compared to the reference Glucose (*P* = 0.021). Rose hip also showed a lower IAUC compared to Glucose but did not reach statistical significance (*P* = 0.086). In comparison to Bilberry and Rose hip, Beetroot had a significantly higher value of IAUCs (*P* = 0.01 and *P* = 0.035, respectively). In the period of 0–90 min, a significant difference was found among the six groups (P = 0.02). Pairwise comparisons showed that except for the Beetroot, the other four probiotic fruit drinks had significantly lower IAUCs compared to the reference Glucose (Bilberry vs. Glucose, *P* = 0.006; Blackcurrant vs Glucose, P = 0.03; Mango vs. Glucose, P = 0.03; and Rose hip vs. Glucose, P = 0.03). In the period of 0–120 min, no significant difference was found among the six groups (*P* = 0.07).

**Table 2:** Incremental area under two-hour serum insulin response curve

Tested products	IAUC(0–15 min) (nmol min/L)	IAUC(0–30 min) (nmol min/L)	IAUC(0–45 min) (nmol min/L)	IAUC(0–60 min) (nmol min/L)	IAUC(0–90 min) (nmol min/L)	IAUC(0–120 min) (nmol min/L)
Glucose (*n* = 11)	45.0 (30.0–82.5)^a^	245.6 (191.3–333.8)^ac^	502.5 (465.0–622.5)^a^	735.0 (706.6–930.0)^a^	907.5 (807.1–1140.1)^a^	907.5 (807.2–1155.9)^a^
Bilberry (*n* = 10)	22.5 (15.0–45.0)^a^	157.1 (104.1–210.0)^b^	340.4 (292.5–405.0)^b^	465.0 (405.9–562.5)^b^	505.0 (457.5–915.0)^b^	598.1 (477.5–1020.0)^a^
Blackcurrant (*n* = 10)	7.5 (0–45.0)^a^	201.9 (79.5–322.5)^abc^	368.0 (279.0–600.0)^abc^	507.2 (450.8–600.0)^abc^	645.4 (592.5–877.5)^b^	697.5 (592.5–907.5)^a^
Beetroot (*n* = 11)	40.5 (18.0–60.0)^a^	234.0 (183.8–363.8)^ac^	525.0 (446.3–652.5)^ac^	684.4 (645.0–764.3)^ac^	900 (705.2–952.5)^ab^	998.5 (846.5–1083.8)^a^
Mango (*n* = 10)	67.5 (0–90.0)^a^	285 (112.9–427.5)^c^	465.0 (320.2.0–810.0)^abc^	583.9 (487.5–1095.0)^abc^	684.1 (517.5–1202.6)^b^	684.1 (532.5–1210.1)^a^
Rose hip (*n* = 11)	7.5 (0–26.3)^a^	180 (81.3–210.0)^b^	375.0 (253.8–551.3)^b^	682.5 (335.7–750.0)^abc*^	801.1 (441.9–993.8)^b^	765.0 (593.3–1068.8)^a^

Superscripted data are presented as median (IQR). Groups that do not share the same letter are significantly different. ^*^only different from Beetroot not Glucose

### Glycemic and insulin indices

Glycemic and Insulin indices were calculated using IAUCs at 90 and 120 min (Table 3). The five test products did not have significantly different glycemic indices compared to the reference Glucose at either time duration. Insulin indices calculated at 90 min showed that Bilberry and Rose hip had significantly lower value than the reference Glucose (P = 0.01 and *P* = 0.012, respectively). Blackcurrant also had significantly lower insulin index compared to Glucose (*P* = 0.037), however, after *P*-value adjustment the difference was no longer significant (*P* = 0.06). Comparisons of insulin indices that were calculated at 120 min showed that Bilberry and Rose hip had significantly lower values than the reference Glucose (*P* = 0.02 and P = 0.02, respectively).

**Table 3:** Glycemic and insulin indices

Tested products	Glycemic index (0–90 min) (%)	Glycemic index (0–120 min) (%)	Insulin index (0–90 min) (%)	Insulin index (0–120 min) (%)
Glucose (*n* = 11)	100	100	100^a^	100^a^
Bilberry (*n* = 10)	88.9 (66.0–101.3)	95.3 (70.6–102.1)	65.7 (54.1–86.7)^b^	63.8 (56.5–86.7)^b^
Blackcurrant (*n* = 10)	75.1 (45.1–141.1)	75.4 (45.1–141.1)	73.8 (61.2–101.6)^b#^	76.9 (59.2–105.0)^ab^
Beetroot (*n* = 11)	85.9 (39.7–112.4)	91.0 (39.1–113.7)	86.9 (74.7–112.9)^ab^	86.5 (73.2–114.6)^ab^
Mango (*n* = 10)	68.4 (43.2–128.9)	68.4 (43.2–129.1)	67.6 (61.2–89.6)^ab^	68.9 (59.3–89.6)^ab^
Rose hip (*n* = 11)	63.5 (41.1–111.3)	58.2 (41.1–112.6)	72.3 (58.1–87.7)^b^	70.8 (62.6–93.6)^b^

Superscripted data are presented as median (IQR). Groups that do not share the same letter are significantly different. ^#^Before *P* value adjustment *P* = 0.034, after adjustment *P* = 0.062

### Polyphenol profiles

The analysis of phenolics (Table 4) showed that the highest values of the total phenolic compounds (280 nm) and flavonols (350 nm) were found in Bilberry whereas Mango had the lowest. The highest values of total anthocyanins/betalains (520 nm) were found in Bilberry and Beetroot, while these families of polyphenols were not detected in either mango or rose hip. Chromatograms corresponding to the HPLC-DAD analysis of the five test products are presented in supplementary figures (see Additional file 1: Figure S1 to S5). Regarding the analysis of the total phenolic measured by FC assay and total antioxidant capacity measured by DPPH and TEAC assays (Table 5), Rose hip beverage was the one with the highest phenolic content and the highest antioxidant capacity. Mango presented the lowest phenolic content and antioxidant capacity.

**Table 4:** Quantification of polyphenol contents by HPLC

Products	Total phenolic compounds (280 nm) (mAU^*^min)	Total flavonols (350 nm) (mAU^*^min)	Total anthocyanins (520 nm) (mAU^*^min)
Bilberry	74.34 (7.70)	13.59 (9.73)	89.56 (9.07)
Blackcurrant	47.29 (7.66)	8.69 (8.0)	30.98 (9.15)
Beetroot	43.40 (4.36)	6.69 (7.52)	74.6 (6.89)
Mango	23.18 (7.17)	0.8 (6.42)	ND^#^
Rose hip	26.35 (6.32)	3.56 (4.27)	ND^#^

Values are presented as Mean (relative standard deviation %). ^#^ND means not detected

**Table 5:** Quantification of phenolic content and total antioxidant capacity

Products	FC assay (mg gallic acid/ml drink)	TEAC assay (mmol trolox/l)	DPPH scavenging activity (%) (dilution factor)
Bilberry	0.41 ± 0.03	16.38 ± 0.68	74.2 ± 3.2 (4)
Blackcurrant	0.61 ± 0.04	45.55 ± 2.0	41.2 ± 1.8 (100)
Beetroot	0.4 ± 0.01	11.22 ± 0.94	54.2 ± 1.4 (4)
Mango	0.18 ± 0.03	8.48 ± 0.39	52.7 ± 1.6 (4)
Rose hip	1.37 ± 0.22	137.9 ± 13.84	76.9 ± 3.0 (100)

Values are presented as Mean ± SD

### Multivariate data analysis with PCA and OPLS-DA

**Fig. 3:**
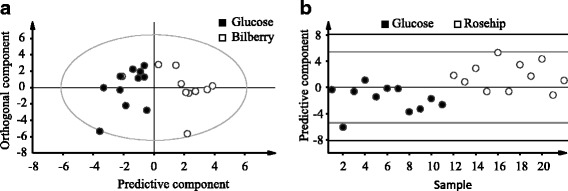
Orthogonal projections to latent structures discriminant analysis (OPLS-DA) score plots of postprandial responses data. **a** Model calculated to discriminate between Bilberry and Glucose (described response variation, R^2^Y = 0.63; predicted response variation, Q^2^Y = 0.48). **b** Model discriminating between Rose hip and Glucose (R^2^Y = 0.43, Q^2^Y = 0.32). The x-axis in (**b**) consists of the 22 analyzed samples. Grey dashed line shows 1.04376*(±2SD) and the black dashed line 1.04376*(±3SD)

**Fig. 4:**
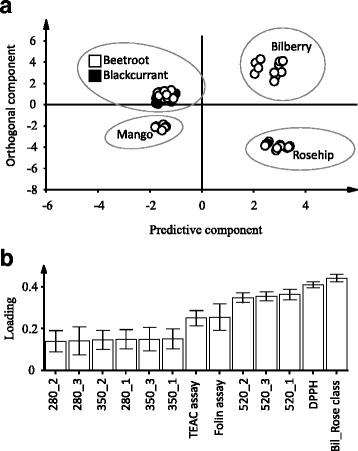
Orthogonal projections to latent structures discriminant analysis (OPLS-DA) model calculated on polyphenol profiles and discriminating between Bilberry and Rose hip (Bil_Rose class) and other tested beverages (described response variation, R^2^Y = 0.977; predicted response variation, Q^2^Y = 0.973). The difference between the two classes of berries was explained by 31.6% of the variation in polyphenol data. **a** Score scatter plot. **b** Loadings bar plot showing the driving forces for the observed cluster separation. The Bil_Rose class had higher levels of phenolics as compared to the other berries

Among the five tested drinks, Bilberry and Rose hip formed clusters separated from the Glucose when analyzing the data of postprandial responses using OPLS-DA (Fig. 3). The pronounced differences in polyphenol profiles of the five drinks were revealed by PCA analysis (Additional file 2: Figure S6). Based on the distinct clusterings of Bilberry and Rose hip that were observed with postprandial responses data and phenolics contents, we performed OPLS-DA by setting Bilberry and Rose hip as one class (Bil_Rose) and the other three products as the second class. The difference between the two classes was explained by 31.6% of the variance (Fig. 4a) and Bil_Rose class had higher levels of phenolics and antioxidant capacities (Fig. 4b).

## Discussion

Consumption of the polyphenol rich probiotic fruit and vegetable beverages did not affect postprandial glycemic responses, but the four fruit drinks supplemented with bilberry, blackcurrant, mango and rose hip significantly attenuated the insulin response in the early postprandial period (0–90 min).

Among the four fruit drinks, Bilberry showed the most profound insulin lowering effect at the 30 min postprandial and this effect was maintained throughout the early postprandial period. Rose hip showed similar efficiency in attenuating postprandial insulin response. Except for the postprandial period 0–60 min, Rose hip led to a decreased insulin response during the 30, 45 and 90 min of postprandial period. GI calculated at 90 and 120 min postprandial were not significantly different between the groups. However, Bilberry and Rose hip had significantly lower II compared with Glucose during both time periods (Table 3). These results from Bilberry and Rose hip are in agreement with our previous study [14], where we found that the bilberry drink attenuated postprandial insulin response in two separate studies and the same rose hip drink showed an insulin attenuating tendency. In addition, in the present study, Bilberry and Rose hip showed higher efficiencies in lowering the early phase insulin responses in comparison to the other test products. This was linked to their higher levels of the phenolic contents (Fig. 4). The different efficiencies in improving postprandial responses have earlier been observed between blackcurrants and lingonberries that containing different subclasses of polyphenols [10].

In the present study, Blackcurrant also led to a reduced early phase postprandial insulin response with a moderate rate compared to Bilberry and Rose hip. At the same time, Bilberry had highest amounts of phenolics, followed by the Blackcurrant (Table 4). Anthocyanins account for the major polyphenols in bilberries [22]. It has been reported that consumption of a bilberry extract containing 36% (*w*/w) anthocyanins, reduced both postprandial glycemic and insulin responses in the individuals with type 2 diabetes [23]. Furthermore, anthocyanins have been reported to be capable of modulating insulin and AMP-activated protein kinase (AMPK) signaling pathways [24, 25]. Thus, a lower level of anthocyanins in Blackcurrant may have contributed to the different efficacies in reducing insulin response. On the other hand, the observed effect from Rose hip indicates that anthocyanins could not be the sole bioactive compound accounting for the attenuated insulin response. Since the seeds were removed from the rose hip fruits, Rose hip drink did not contain any anthocyanins. On the other hand, Rose hip had the highest amount of total phenolic content. It should be noted that FC assay reagent is nonspecific to phenolic compound, instead it measures the total antioxidant capacity. Therefore, high amount of vitamin C and carotenoids in Rose hip [26, 27] could have contributed to the observed highest antioxidant capacity. The highest antioxidant capacity of Rose hip was also confirmed by the TEAC and DPPH assays. Rose hips have been used in traditional medicine for treating multiple illnesses [28]. Consumption of the rose hip extract significantly reduced hyperglycemia in diabetic rats. It was speculated that the antioxidant property of the rose hip extract led to a reduction in the oxidative stress, which helped preserving the pancreatic β-cell integrity and finally led to insulinotrophic action [29]. In a recent study in vitro, rose hip extract enhanced the pancreatic β-cell proliferation [30]. In an animal study, it was reported that intake of rose hip extract led to increased insulin sensitivity via downregulation of hepatic lipogenesis [31]. Although the bioactive compounds in rose hip contributing to the observed effects were not identified, these findings suggest that rose hip could have multiple mechanisms of action leading to an improved insulin response.

Interestingly, despite the lowest values determined for total phenolic content, total flavonols and total antioxidant capacity in Mango we observed a decreased postprandial insulin response at 90 min. It has been reported that a major phenolic compound in mango, mangiferin [32] could improve glucose and insulin profiles in high fat fed mice [33]. Also, other bioactive compounds such as carotenoids, vitamin C and dietary fiber in mango may affect the blood glucose and insulin metabolism [34]. On the contrary, the high polyphenol content in Beetroot was not associated with such a beneficial effect. Again, this indicates that the polyphenol content or antioxidant capacity itself could not be directly linked to the insulin attenuating effects of the present test products. To note, Beetroot had the second highest value of anthocyanin content, which should be from the betalains (pigment compound of beetroot) sharing structural similarities with anthocyanins. Although the retention times of the peaks from Beetroot were different from those of Bilberry and Blackcurrant, we could not distinguish between anthocyanin and betalain with the current HPLC analysis settings due to lack of standard and subsequent mass spectrometry analysis for identification. A degradation product of betalain, neobetanin was associated with a reduced early phase postprandial (0–60 min) insulin response [9]. However, in the present study we did not observe beneficial effects from Beetroot. We postulated that the postprandial glycemic and insulin improving effects could be linked to the polyphenol content. The high efficiency of Bilberry and Rose hip in attenuating early postprandial insulin responses were correlated with higher phenolic contents. However, phenolic levels alone were not directly linked to the insulin lowering effect. It cannot be ruled out that specific phenolic components could serve as the contributing factors for the observed insulin attenuating effect in the four fruit supplemented test products. Moreover, other bioactive compounds present in the test products may have contributed in a synergistic manner when decreasing insulin demand.

There are limitations in the current study that could be improved in the future research. We intended to investigate and compare the effects of test products with a baseline product containing only fermented oatmeal besides the glucose control group. Unfortunately, we had to exclude the fermented oatmeal group from the data analysis due to improper carbohydrate compensation, and could not assess the impact of probiotic fermented oatmeal per se. Although not evaluated in the current study, beneficial effects of probiotic bacteria in managing glycemic control have been demonstrated in multiple animal models and human studies [35, 36]. Further research efforts are needed to discover the underlying mechanisms.

## Conclusions

Consistent with the previous study [14], we found an inconsistency between the early glycemic and the insulin responses. The intake of test products containing bilberry, rose hip, blackcurrant or mango did not affect the postprandial glycemic response, but attenuated early stage postprandial (0–90 min) insulin responses. The higher efficiency of bilberry and rose hip drinks in lowering insulin responses was associated with amounts of phenolics. The health-related relevance of our findings is that consumption of food products that induce a lower insulin response may enhance insulin sensitivity.

## Additional files

Additional file 1: **Figure S1.** Chromatograms corresponding to the HPLC-DAD analysis of bilberry beverage at 280 nm, 350 nm and 520 nm. P, phenolic compounds; F, flavonols; and A, anthocyanins. **Figure S2.** Chromatograms corresponding to the HPLC-DAD analysis of blackcurrant beverage at 280 nm, 350 nm and 520 nm. P, phenolic compounds; F, flavonols; and A, anthocyanins. **Figure S3.** Chromatograms corresponding to the HPLC-DAD analysis of Rose hip beverage at 280 nm, 350 nm and 520 nm. P, phenolic compounds; and F, flavonols. **Figure S4.** Chromatograms corresponding to the HPLC-DAD analysis of Mango beverage at 280 nm, 350 nm and 520 nm. P, phenolic compounds; and F, flavonols. **Figure S5.** Chromatograms corresponding to the HPLC-DAD analysis of beetroot beverage at 280 nm, 350 nm and 520 nm. P, phenolic compounds; F, flavonols; and B, betalains. (DOCX 2460 kb)

Additional file 2: **Figure S6.** PCA score plot of data from phenolic analysis of the five tested drinks. PC1 and PC2 explained 60.4 and 25.2% of the variance in the data respectively. (PDF 49 kb)
