# Supplementary material for: Probiotic fruit beverages with different polyphenol profiles attenuated early insulin response
Source: Nutr J. 2018 Feb 27;17:34. doi: 10.1186/s12937-018-0335-0 (PMC5827978; doi:10.1186/s12937-018-0335-0)
Supplement: Supplementary file 1 — Figure S1. Chromatograms corresponding to the HPLC-DAD analysis of bilberry beverage at 280 nm, 350 nm and 520 nm. P, phenolic compounds; F, flavonols; and A, anthocyanins. Figure S2. Chromatograms corresponding to the HPLC-DAD analysis of blackcurrant beverage at 280 nm, 350 nm and 520 nm. P, phenolic compounds; F, flavonols; and A, anthocyanins. Figure S3. Chromatograms corresponding to the HPLC-DAD analysis of Rose hip beverage at 280 nm, 350 nm and 520 nm. P, phenolic compounds; and F, flavonols. Figure S4. Chromatograms corresponding to the HPLC-DAD analysis of Mango beverage at 280 nm, 350 nm and 520 nm. P, phenolic compounds; and F, flavonols. Figure S5. Chromatograms corresponding to the HPLC-DAD analysis of beetroot beverage at 280 nm, 350 nm and 520 nm. P, phenolic compounds; F, flavonols; and B, betalains. (DOCX 2460 kb) [file 12937_2018_335_MOESM1_ESM.docx]

**Additional file 1**

**Supplementary Figure S1.** Chromatograms corresponding to the HPLC-DAD analysis of bilberry beverage at 280 nm, 350 nm and 520 nm. P, phenolic compounds; F, flavonols; and A, anthocyanins.

**Supplementary Figure S2.** Chromatograms corresponding to the HPLC-DAD analysis of blackcurrant beverage at 280 nm, 350 nm and 520 nm. P, phenolic compounds; F, flavonols; and A, anthocyanins.

**Supplementary Figure S3.** Chromatograms corresponding to the HPLC-DAD analysis of Rose hip beverage at 280 nm, 350 nm and 520 nm. P, phenolic compounds; and F, flavonols.

**Supplementary Figure S4.** Chromatograms corresponding to the HPLC-DAD analysis of Mango beverage at 280 nm, 350 nm and 520 nm. P, phenolic compounds; and F, flavonols.

**Supplementary Figure S5.** Chromatograms corresponding to the HPLC-DAD analysis of beetroot beverage at 280 nm, 350 nm and 520 nm. P, phenolic compounds; F, flavonols; and B, betalains.
