# Supplementary figures and images for: Probiotic fruit beverages with different polyphenol profiles attenuated early insulin response
Source: Nutr J. 2018 Feb 27;17:34. doi: 10.1186/s12937-018-0335-0 (PMC5827978; doi:10.1186/s12937-018-0335-0)

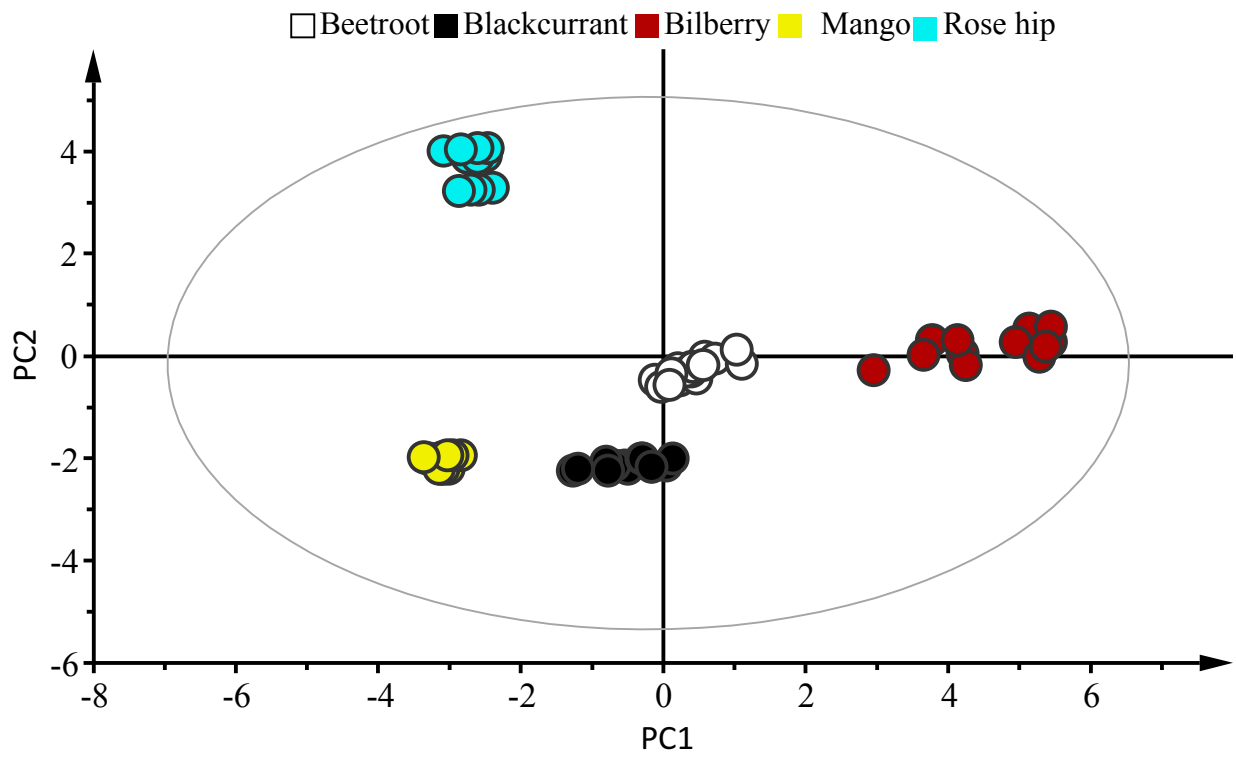

Supplement: Supplementary file 2 — Figure S6. PCA score plot of data from phenolic analysis of the five tested drinks. PC1 and PC2 explained 60.4 and 25.2% of the variance in the data respectively. (PDF 49 kb) [file 12937_2018_335_MOESM2_ESM.pdf]
